# Genome-wide association study for polledness, horn shape, and wool traits in Original Valachian sheep

**DOI:** 10.5194/aab-67-373-2024

**Published:** 2024-07-30

**Authors:** Mária Mészárosová, Gábor Mészáros, Nina Moravčíková, Ivan Pavlík, Milan Margetín, Radovan Kasarda

**Affiliations:** 1 Institute of Nutrition and Genomics, Slovak University of Agriculture in Nitra, Tr. A. Hlinku 2, 94976 Nitra, Slovakia; 2 BOKU University, Gregor-Mendel-Straße 33, 1180 Vienna, Austria; 3 NAFC – Research Institute for Animal Production Nitra, Hlohovecká 2, 95141 Lužianky, Slovakia

## Abstract

The Original Valachian sheep is an endangered Slovak national breed that is well adapted to high-altitude pastures. The sheep can be horned with various shapes and can have multi-coloured or completely white or black wool. Breeders are interested in learning about the genetic basis of these traits. We conducted a genome-wide association study based on the genomic information of 96 sheep genotyped by the GeneSeek GGP Ovine 50K SNP (single-nucleotide polymorphism) chip and on the following traits: polledness (presence or absence of horns), horn shape, and wool colour (completely white and completely black). The univariate linear mixed model was used to discover genetic variants significantly associated with tested traits. The Bonferroni correction and the false-discovery rate were used as significance thresholds. The *RXFP2* gene (chromosome 10, 29.5 Mb) was identified as a strong candidate for polledness. In addition, when compared to animals with sideways-turned horns vs. polled, the region around the *ADAMTS3* gene (chromosome 6, 88.47 Mb) was significant. A total of nine significant genomic regions were found when comparing the sideways-turned spiral horns with the backwards-curled horns, the two most frequent horn types in Original Valachian sheep. The *RXFP2* may also contribute to the genetic control of horn shape. Genes identified in other regions were involved to osteogenic differentiation and osteoblast proliferation (*PCP4*, chromosome 1, 260.7 Mb), bone mineral density and mineral content (*NKX1-2*, chromosome 22, 43.75 Mb). The significant genetic variants close to the region of *MC1R* (chromosome 14, at 14.2 Mb) were associated with the wool colour of sheep that were fully white or fully black animals. The results of this study will contribute to a better understanding of the phenotypic variability of the Original Valachian sheep, especially regarding traits that are very important for breeders of this endangered breed.

## 1 Introduction

Domestication of sheep led to a wide diversity of traits. Among such traits, which are also relevant for agriculture, we have the coat colour, wool, horns, tail, ears, and udder. The availability of high-density genotype data in the last decade paved the way towards investigating the genetic determination of these traits in sheep (Kalds et al., 2022).

The Original Valachian sheep currently bred in Slovakia have their origins in the historical Valachian culture, which spread across central Europe during the Middle Ages. Valachian sheep arrived in the territory of present-day Slovakia with the Valachian colonisation in the 13th and 14th centuries. Valachian sheep were traditionally bred for generations at altitudes of 600 to 1200 m, mainly in the mountain regions of northern Slovakia. Over centuries, these sheep adapted to local conditions and gradually evolved into the breed known today as the Original Valachian sheep. Breeds that are phenotypically similar to Original Valachian sheep are also kept in other European countries, including the Czech Republic (Vlachian sheep), Poland (Polish Mountain sheep), the Balkan countries (Zackel or Vlaska), Romania (Tsurcana), Bulgaria (Karakachan), or Ukraine (Ukrainian Mountain sheep) (Drăgănescu and Grosu, 2010). Today, the Original Valachian sheep is recognised as a Slovak national breed that is well adapted to the high-altitude regions and pastures in the northern part of Slovakia. It is a triple-purpose breed that produces milk, meat, and wool. As the main interest was always in the breed's adaptability, there was no selection based on phenotypic uniformity in the past. Thus, the Original Valachian sheep is characterised by various coat colour variations and horn types. Because the production efficiency of Original Valachian sheep is lower compared to other commercial breeds and because the pasture systems in high mountains are not used as frequently as in the past, this breed became endangered. The recent population size was about 3600 animals, with 300 females in 10 nucleus farms. From the phenotypic traits, the main interest of breeders is in horn traits (presence or absence), the horn shape, and wool quality and colour. From the evolutionary perspective, the horns in sheep act as weapons, shields, shock absorbers, or display organs to intimidate opponents when competing for mate pairs and as visual dominance rank symbols (Geist, 1966). While the presence of horns is considered to be an advantage in nature, the hornless, so-called “polled” animals are favoured in livestock breeding. The reason for favouring hornless animals is to reduce safety risks for farmers and to increase the welfare of other animals in the flock (Simon et al., 2022). In connection to polledness and horn shape, the *RXFP2 * gene was highlighted as the main driver behind horn development (Kalds et al., 2022). The polled phenotype in sheep is often linked to a single locus, a 1.78 kb insertion, in the *RXFP2* gene region (Duijvesteijn et al., 2018). This mutation was also found to be responsible for about 76 % of the additive genetic variation in horn size (Johnston et al., 2011). While *RXFP2* is associated with horn status, sex also influences horn growth in some breeds (Duijvesteijn et al., 2018). Duijvesteijn et al. (2018) suggested that there might be another causal genetic variant influencing polledness in sheep, although no others have been identified so far. Wang et al. (2019) identified 624 other genes via sequencing and transcriptome analysis that could be related to horn development in deer, goats, and sheep. Some of them, namely *SOX10*, *SNAI1*, and *TFAP2A*, were expressed in fetal horn buds but not in the adjacent skin of the studied animals. L. Chen et al. (2019), using transcriptomic data from the horn sprouts of sheep and goats, found a relationship between the expression of genes such as *KRT1*, *KRT2*, *KRT3*, and other keratin-related genes and horn development. Interestingly, the polled genotype is already widespread in various sheep breeds, with many breeds being completely hornless. Horns occur mainly in autochthonous breeds (Simon et al., 2022), such as Original Valachian sheep kept in Slovakia.

The characteristics of the sheep wool are among the defining features of sheep breeds. One of the most important characteristics is the wool colour. In sheep, the genes related to coat colour are *ASIP*, *MC1R*, and *TYRP1* (Parsons et al., 1999; Våge et al., 1999; Beraldi et al., 2006). The *MC1R* gene was specifically studied concerning the black coat colour in Chinese sheep breeds (Yang et al., 2013). Both synonymous and non-synonymous mutations in the *MC1R* gene were found to significantly affect fully white or black colours in different sheep breeds. A single haplotype in the *MC1R* gene was uniquely associated with the black colour in the Minxian black-fur breed (Yang et al., 2013). The *MC1R*, *ASIP* (Smit et al., 2002; Fontanesi et al., 2011), and *MSH* (Klungland et al., 1995) genes regulate the amount and proportion of pigment resulting in the base coat colours of red and black. From other genes, *KIT* and *MITF* (Li et al., 2014) were found to be involved in the genetic control of coat colour in sheep. The coat colour variations and their genetic background, specifically for sheep, were also reviewed by Koseniuk et al. (2018) and Kalds et al. (2022). Both reviews concluded that, although many genes influence coat colour in sheep, there is still no conclusive evidence of established polymorphisms for specific coat colour types.

In this paper, we address a gap in knowledge by analysing three traits not yet explored in the Original Valachian sheep population: polledness and wool colour. We hypothesise that the polledness and colour traits are influenced by a few major genes. In the Original Valachian sheep population, there is no selection on a specific horn type; therefore, there is variation in this trait, with the sideways and the backwards-curled shapes being the most common. Both sexes of the Original Valachian sheep grow horns.

## 2 Material and methods

### 2.1 Genome-wide data and analysed traits

Genome-wide data of 96 autochthonous Original Valachian sheep (78 females and 18 males – representative samples from multiple herds) were analysed in this study. The animals were genotyped using the GeneSeek GGP Ovine 50K SNP (single-nucleotide polymorphism) chip. The quality controlling and the data preparation for genome-wide association studies (GWAS) analyses were done using PLINK 1.9 (Chang et al., 2015). Within the quality controlling, animals and SNPs with more than 10 % missing genotypes, SNPs with less than 5 % minor allele frequency (maf), and all SNPs not on the autosomes were removed. After the quality controlling, 38 466 SNP variants and all 96 animals remained. Average genomic relatedness between genotyped animals was 0.06 (median: 0.02, standard deviation: 0.08) and was derived from the IBD matrix calculated by PLINK – genome option (Chang et al., 2015).

The horn phenotypes were defined in two different ways: the polledness (presence or absence of horns) and horn shape. At the time of sampling, the animals were 2 to 6 years old; thus, any horn phenotype could be clearly distinguished. A description of the horn phenotypes analysed in this study, the local original Slovak terms, and the number of animals with the particular horn phenotype are shown in Table 1. The horn phenotypes were available for 90 sheep.

**Table 1:** Horn types in Original Valachian sheep.

	Horn type (original	Description	Number of
	expression in Slovak)		observations
1	Sideways horns (“širaňa”)	lyre-shaped sideways and spirally curled horns	47
2	Backwards-curled horns (“saňa”)	backwards-curled horns, similar to most Ibex-type goats	12
3	Reduced horns (“kymla”)	significantly reduced horns, sometimes only to a few centimetres	4
4	Polled (“šuta”)	polled sheep without horns	24
5	Non-identified horn type (“kornuta”)	horns without any further classification	3

The Original Valachian sheep is characterised by a wide variety of colours and colour combinations. Therefore, we divided animals into three colour categories: completely white coloured ( n=18 ), completely black coloured ( n=13 ), and sheep with a variety of coat colours ( n=59 ). Six sheep were omitted from the analysis due to no recorded phenotype for the wool colour.

### 2.2 Genome-wide association analysis

Six separate GWAS runs were performed: three for polledness (polled vs. non-polled animals, sideways-horned vs. polled animals, backwards-curled horns vs. polled animals), one for horn shape (backwards-curled vs. sideways-horned animals), one for wool quality, and one for coat colour (white coloured vs. black coloured animals). The sex and the herd were tested as fixed effects using one-way ANOVA. The effect of sex was significant ( p=0.0133 ) for the horn traits. Therefore, it was included in the GWAS models for horn phenotypes. The GWAS was performed using GEMMA (Zhou et al., 2012) based on the univariate linear mixed model in Eq. (1):

1 $$\begin{array}{rl} & y=W\alpha +x\beta +\epsilon ;\;u∼{\mathrm{MVN}}_{n}\left(0,\;\lambda \tau^{-1}K\right),\\ & \epsilon ={\mathrm{MVN}}_{n}\left(0,\;\tau^{-1}I_{n}\right), \end{array}$$

where y is an n vector of phenotypes for the sheep; W is the matrix of covariates (fixed effects), in our case consisting of a column of 1 s and the effect of sex; α is a c vector of the corresponding coefficients, including the intercept; x is an n vector of marker genotypes; β is the effect size of the marker and is an estimate of the marker–SNP additive effect; ϵ is an n vector of errors; τ -1 is the variance of the residual errors; λ is the ratio between the two variance components; K is a known n×n relatedness matrix; and In is an n×n identity matrix. MVN ${}_{n}$ denotes the n -dimensional multivariate normal distribution. The correction for population structure, an important step to achieve correct GWAS results (Price et al., 2010), was based on the centred relatedness matrix K calculated within GEMMA. Two statistical approaches were subsequently used for correction for multiple testings, namely the Bonferroni correction (Bonferroni, 1936) and the local false-discovery rate (Efron, 2010). The Bonferroni correction was calculated following Eq. (2):

2 $$p_{\mathrm{Bonferroni}}=\frac{0.05}{n_{\mathrm{SNP}}},$$

where $p_{\mathrm{Bonferroni}}$ is the new p -value threshold after the Bonferroni correction, 0.05 is the initial p -value threshold for significance, and $n_{\mathrm{SNP}}$ is the number of SNPs in the GWAS analysis.

For the practical computation of the local false-discovery rate (FDR), we used the locfdr R package (version 1.1-8), with a threshold in terms of the z score set to 0.2 according to the recommendations of Efron (2010) and Waldmann et al. (2013). The z score is a statistical metric to measure the distance of any given value from the mean. FDR was calculated separately for each GWAS run.

The significant regions identified by GWAS were explored for gene content using the web-based tool the Genome Data Viewer of the National Center for Biotechnology Information (NCBI, https://www.ncbi.nlm.nih.gov/genome/gdv/, last access: 7 January 2024), based on the actual genome assembly ARS-UI_Ramb_v2.0, and for quantitative trait loci (QTL) content using the sheep QTLdb database (https://animalgenome.org/QTLdb/, last access: 7 January 2024). The biological function of detected genes was assessed based on previous studies, considering only genes with potential influence over the phenotypic traits of interest.

## 3 Results and discussion

### 3.1 Genomic regions influencing horns

#### 3.1.1 Polledness

**Figure 1:**
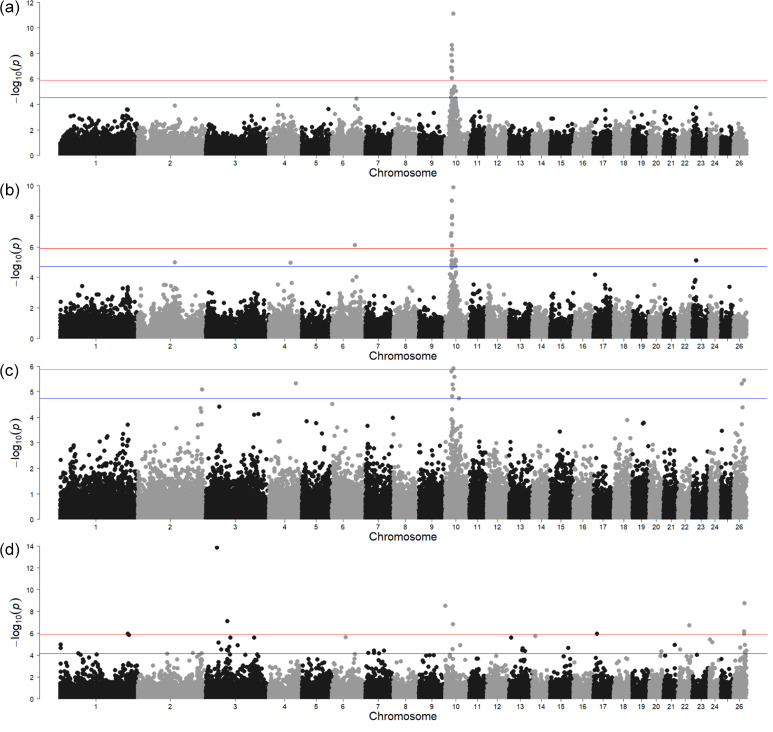
Genome-wide associations for horn traits. For SNP significance, the red line indicates the Bonferroni threshold, and the blue line is below the local FDR threshold. **(a)** A case control study of the polled and non-polled animals; **(b)** a case control study of sideways-horned and polled animals; **(c)** a case control study of animals with backwards-curled horns and polled animals; **(d)** a direct comparison of backwards-curled and sideways-horned animals.

The GWAS for polled vs. horned animals resulted in one strong signal above the Bonferroni threshold (-log10 (p)=5.90 ) and another with the local FDR (-log10 (p)=4.54 ) (Fig. 1a, Table S1 in the Supplement). In a relatively wide region on chromosome 10 (between 23.18 and 30.90 Mb), several significant SNPs (-log10 (p)=7.85 ) were located. Of the protein-coding genes in this region, the most interesting was *RXFP2* (chromosome 10, 29.5 Mb) in sheep previously associated with polledness (Wiedemar and Drögemüller, 2015) and spiral and horizontally extended horn shapes (Pan et al., 2018). Moreover, as shown by Wiedemar et al. (2014), this gene may have a similar function between different livestock species as its overexpression, together with FOXL2 in horn buds, has also been shown in cattle. This suggests that the *RXFP2* gene is a promising candidate for sheep polledness. However, the identification of causal genetic variants, which are usually breed-specific, requires further research focused on sequencing the gene region in different sheep breeds.

The second region, very close to the local FDR threshold, was identified on chromosome 6 (-log10 (p)=4.46 ) at 93.37 Mb. The *MRPL1* gene located close to this signal was also detected by a previous transcriptome analysis study comparing Poll Dorset and short-tailed Hans Sheep (Liu et al., 2015). This study was not specific to the analysis of polled phenotypes but focused instead on the differences in gene expression from 60, 90, and 120 d fetal skeletal muscle. Nevertheless, the fact that the expression of the *MRPL1* gene was found only in the non-polled breed makes it an interesting candidate. Although the number of animals in our analysis of the horn traits i was low, the *MRPL1* gene could be investigated further This corresponds with the suggestion of Simon et al. (2020) that there could be other causal genes for the polled phenotype.

#### 3.1.2 Sideways-horned vs. polled animals

The GWAS of animals with sideways horns ( n=47 ) and polled animals ( n=24 ) resulted in two signals above the Bonferroni threshold (-log10 (p)=5.90 ) and two above the local FDR (-log10 (p)=4.71 ) (Fig. 1, Table S1). The most significant was located on chromosome 10 near *RXFP2*. The second region, denoted by only a single SNP above the Bonferroni threshold, was on chromosome 6 at 88.76 Mb. One of the interesting protein-coding genes in this region was the *ADAMTS3* gene (chromosome 6, 88.47 Mb). Many genes of the ADAMTS family, to which the *ADAMTS3* gene belongs, were also found to be highly expressed in the antlers and headgear of ruminants (Wang et al., 2018). This gene is involved in collagen maturation and biosynthesis and had an influence on osteosarcoma in human cell lines (Aydemir et al., 2018).

The local FDR threshold further identified three genomic regions on chromosome 2 (143.33 Mb), chromosome 4 (83.45 Mb), and chromosome 23 (14.93 Mb). The three genes closest to the signal on chromosome 2 were from the same gene family (*SCN7A*, *SCN9A*, and *SCN1A*) but without apparent connection to polledness or any similar phenotype. The closest to the signal on chromosome 4 was a transcription-factor-2-like protein-coding gene (*LOC121819513*) which was not characterised yet. The two other genes within 0.5 Mb up- and downstream of the genome were *POU6F2* and *VPS41* but with no connection to traits related to horns or bones. Similarly, within the vicinity of the signal on chromosome 23 Mb (14.93 Mb) (maf = 0.11), there were no protein-coding genes.

#### 3.1.3 Animals with backwards-curled horns vs. polled animals

The animals with backwards-curled horns ( n=12 ) were compared to polled animals ( n=24 ) (Fig. 1c) and resulted in only one signal above the Bonferroni (-log10 (p)=5.90 ) and local FDR (-log10 (p)=4.75 ) thresholds. While the Bonferroni threshold was the same for all GWAS analyses, the local FDR threshold was individual to each analysis – in this case, even stricter than the Bonferroni correction. Also, here, the most significant signal was on chromosome 10 (at 31.29 Mb), albeit somewhat shifted from the location of the *RXFP2* gene (at 29.5 Mb). There are several other SNPs (at 28.5 and 29.6 Mb) in the immediate vicinity that are above the FDR threshold; thus, the signal picked up SNPs that are in high linkage disequilibrium (LD) with the *RXFP2* gene. In the region around the most significant SNP on chromosome 10, there were four genes (*UBL3, SLC7A1, MTUS2, SLC46A3*), but none of them seemed to be associated with horn development.

Based on the FDR threshold, there were four other significant regions: on chromosome 2 in position 247.47 Mb (maf = 0.46), on chromosome 4 in position 103.41 Mb (maf = 0.13), and on chromosome 26 in positions 31.67 Mb (maf = 0.35) and 40.22 Mb (maf = 0.29). Five genes (*UBR4, IFFO2, ALDH4A1, TAS1R2, PAX7*) were located near the signal on chromosome 2. From these genes, the most important seems to be the *PAX7* gene. Although it was primarily associated with muscle development and myogenesis, there is evidence that it is indirectly involved in cranial facial development in humans via the binding protein *PAXBP1* (Blake and Ziman, 2014; Aldersey et al., 2020). There were also five genes (*DGKI, CREB3L2, AKR1D1, TRIM24, SVOPL*) located in the genomic regions around the signal on chromosome 4. *CREB3L2* may be important for horn development in cattle, with different expressions when comparing horn tissues and prenatal tissues (Shil et al., 2017). On chromosome 26, there were four genes (*KCNU1, ZNF703, ERLIN2, PLPBP*) around the most significant SNP at 31.67 Mb and a single gene (*RARB*) around the SNP at 40.22 Mb. None of these genes seem to have any plausible connection to horn development in sheep or other species.

#### 3.1.4 Backwards-curled vs. sideways-horned animals

The GWAS comprising the genomic data of animals with backwards-curled ( n=12 ) and sideways-turned horns ( n=47 ) showed eight regions over the Bonferroni threshold (-log10 (p) = 5.90) and a large number of regions above the local FDR threshold (-log10 (p) = 4.11) (Fig. 1d). Due to a large number of signals with lower significance values, we have focused mainly on the strongest signals identified by the Bonferroni threshold. The positions of the detected genomic regions, including the QTLs and gene occurrence summary, are presented in Table S1.

There were four significant SNPs on chromosome 10 in position 29.45 Mb, with SNP IDs RXFP2_insert_L1, RXFP2_insert_L2, RXFP2_ insert_ R1, and RXFP2_insert_R2 (maf = 0.05 for all). This again indicated that the *RXFP2 * gene could play a significant role in horn shape (Pan et al., 2018) as it was found to be a significant signal in sideways-turned horns (Fig. 1b) and in backwards-curled horns (Fig. 1c).

The signal consisting of one SNP on chromosome 1 (at 260.7 Mb, maf = 0.06) was near the *PCP4* gene, with a proven role in osteogenic differentiation in rat stem cells (Xiao et al., 2008); this refers to the capacity of stem cells to self-renew and differentiate to multiple cell types, including osteocytes. This gene plays an important role also in osteoblast proliferation/differentiation (Meng et al., 2020). This suggests that this gene could also be responsible for the qualitative differences in horn types in Original Valachian sheep. According to Johnston et al. (2011), however, any signals with a minor allele frequency of less than 0.1 in a low number of individuals with phenotypes should be interpreted with care as there is a chance of a false-positive association in terms of the over-representation of rare genotypes.

**Figure 2:**
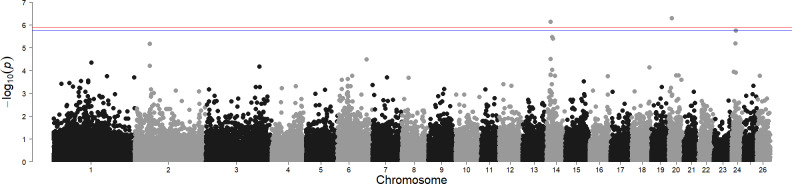
Genome-wide associations for white- and black-coloured sheep. For SNP significance, the red line indicates the Bonferroni threshold, and the blue line is below the local FDR threshold.

There was a significant signal above the Bonferroni threshold on chromosome 22, centred on 43.75 Mb (maf = 0.17). In humans, the gene *NKX1-2* plays a role in differentiating bone marrow mesenchymal precursor cells into adipocytes (fat cells) or osteoblasts and in regulating between them (N. Chen et al., 2019). The gene *ABRAXAS2* was associated with bone mineral density and bone mineral content in humans (Chen et al., 2020). It is not clear how the regulatory activities of these genes would influence the horn type in sheep, but their presence in our results could initiate further research into this matter.

The significant SNPs on chromosome 26 (40.5 to 42.4 Mb) indicated the influence of the *THRB* gene. The thyroid hormone is essential for development and ossification during fetal development and plays an important role in the linear growth and maintenance of bone mass in humans (Bassett and Williams, 2003). This gene was also found to be connected to calcium and phosphate concentration, including the regulation of bone mass (Cardoso et al., 2014).

The genomic regions on chromosome 3 (42.35 Mb (maf = 0.07) and 82.69 Mb (maf = 0.07)), 10 (0.01 Mb (maf = 0.12)), 17 (14.11 Mb (maf = 0.07)), and 26 (42.44 Mb (maf = 0.04)) did not contain genes known to be relevant to horns or bones in any species. Similarly to the previous GWAS herein, the number of animals with backward-curled horns should be increased in the future to confirm the role of identified SNPs and potential candidate genes in genetic controlling of horn shape in sheep.

### 3.2 Genomic regions influencing wool colour

The results for the genome-wide association for wool colour, considering only fully white and fully black animals, identified two significant regions above the Bonferroni threshold (-log10 (p) = 5.90) and another one region above the local FDR threshold (-log10 (p) = 5.75), as shown in Fig. 2. The most prominent signal is on chromosome 14 at 14.23 Mb (maf = 0.13). This region contains the *MC1R* gene (chromosome 14, at 14.2 Mb), regulating the amount and proportion of pigment resulting in red and black base colours in multiple species. In sheep, the *MC1R *gene was also identified for its association with wool pigmentation in Brazilian Creole sheep (Hepp et al., 2012). Rochus et al. (2019) studied this gene specifically for its effect on black colouring. A wide range of studies exist for a wide range of species in livestock, but also for Arabian camels (Almathen et al., 2018), carp (Mandal et al., 2020), or Japanese quail (Li et al., 2019).

Another significant SNP was found on chromosome 20 (10.77 Mb, maf = 0.40). However, from the genes in this region (*STK38, SRSF3, CDKN1A, RAB44, CPNE5, PPIL1, MTCH1, FGD2*), we did not identify any genes directly or indirectly associated with coat colour.

In addition, the local FDR identified another significant SNP on chromosome 24 (15.37 Mb, maf = 0.15). The immediate vicinity of the significant SNP contains only un-characterised genes. The closest protein-coding gene is the *ABCC6* gene at 15 Mb, which does not appear to be connected to colour traits in sheep or any other species.

## 4 Conclusions

A genome-wide association study for polledness and wool traits was conducted in Original Valachian sheep, an autochthonous breed from Slovakia. Obtained results indicated that the main signal for the polled phenotype was in the vicinity of the *RXFP2* gene on chromosome 10, confirming the influence of this gene shown in other studies on different breeds. Other SNPs on chromosome 6, near the *MRPL1* gene, could also be considered to be important genetic variants for polledness in Original Valachian sheep. The horn shape also seems to be influenced by the *RXFP2* gene as this was identified in the associations containing animals with sideways-turned and backwards-curled horns. Furthermore, the region containing this gene was also significant when comparing the animals with different horn types to each other. Even if the *RXFP2* gene was previously identified in other studies, this study showed its significance for the first time in Valachian sheep, which is an important local breed not only in Slovakia but also in neighbouring countries. When comparing fully black and fully white animals, the results confirmed the *MC1R* gene on chromosome 14 as the candidate gene for wool colour. The results of this study will contribute to a better understanding of the phenotypic variability of the Original Valachian sheep, especially regarding traits that are very important for breeders of this endangered breed.

## Supplement

10.5194/aab-67-373-2024-supplement The supplement related to this article is available online at: https://doi.org/10.5194/aab-67-373-2024-supplement.

## Data Availability

The data presented in this study are available on request from the corresponding author. The data are not publicly available due to ongoing research.

## References

[bib1.bib1] Aldersey JE, Sonstegard TS, Williams JL, Bottema CDK (2020). Understanding the effects of the bovine POLLED variants. Anim Genet.

[bib1.bib2] Almathen F, Elbir H, Bahbahani H, Mwacharo J, Hanotte O (2018). Polymorphisms in MC1R and ASIP Genes are Associated with Coat Color Variation in the Arabian Camel. J Hered.

[bib1.bib3] Aydemir AT, Alper M, Kockar F (2018). SP1-mediated downregulation of ADAMTS3 gene expression in osteosarcoma models. Gene.

[bib1.bib4] Bassett JHD, Williams GR (2003). The molecular actions of thyroid hormone in bone. Trend Endocrinol Metab.

[bib1.bib5] Beraldi D, McRae AF, Gratten J, Slate J, Visscher PM, Pemberton JM (2006). Development of a Linkage Map and Mapping of Phenotypic Polymorphisms in a Free-Living Population of Soay Sheep (*Ovis aries*). Genetics.

[bib1.bib6] Blake JA, Ziman MR (2014). Pax genes: regulators of lineage specification and progenitor cell maintenance. Development.

[bib1.bib7] Bonferroni C (1936). Teoria statistica delle classi e calcolo delle probabilita. Pubblicazioni R Ist Super Sci Econ E Commericiali Firenze.

[bib1.bib8] Cardoso LF, de Paula FJA, Maciel LMZ (2014). Resistance to thyroid hormone due to mutations in the THRB gene impairs bone mass and affects calcium and phosphorus homeostasis. Bone.

[bib1.bib9] Chang CC, Chow CC, Tellier LC, Vattikuti S, Purcell SM, Lee JJ (2015). Second-generation PLINK: rising to the challenge of larger and richer datasets. GigaScience.

[bib1.bib10] Chen L, Qiu Q, Jiang Y, Wang K, Lin Z, Li Z, Bibi F, Yang Y, Wang J, Nie W, Su W, Liu G, Li Q, Fu W, Pan X, Liu Ch, Yang J, Zhang C, Yin Y, Wang Y, Zhao Y, Zhang C, Wang Z, Qin Y, Liu W, Wang B, Ren Y, Zhang R, Zeng Y, da Fonseca RR, Wei B, Li R, Wan W, Zhao R, Zhu W, Wang Y, Duan S, Gao Y, Zhang YE, Chen C, Hvilsom C, Epps CW, Chemnick LG, Dong Y, Mirarab S, Siegismund HR, Ryder OA, Gilbert MTP, Lewin HA, Zhang G, Heller R, Wang W (2019). Large-scale ruminant genome sequencing provides insights into their evolution and distinct traits. Science.

[bib1.bib11] Chen N, Schill RL, O’Donnell M, MacDougald OA, Koenig RJ, Xu B (2019). The transcription factor NKX1-2 promotes adipogenesis and may contribute to a balance between adipocyte and osteoblast differentiation. J Biol Chem.

[bib1.bib12] Chen N, Schill RL, O'Donnell M, Xu K, Bagchi DP, MacDougald OA, Koenig RJ, Xu B (2020). Genetic variants affecting bone mineral density and bone mineral content at multiple skeletal sites in Hispanic children. Bone.

[bib1.bib13] Draganescu C, Grosu H (2021). Valachian (Zackel) heritage philetic sheep group – a taxonomic problem. Scientific Papers, Rom Acad.

[bib1.bib14] Duijvesteijn N, Bolormaa S, Daetwyler HD, van der Werf JHJ (2018). Genomic prediction of the polled and horned phenotypes in Merino sheep. Genet Sel Evol.

[bib1.bib15] Efron B (2010). Large-Scale Inference: Empirical Bayes Methods for Estimation, Testing, and Prediction.

[bib1.bib16] Fontanesi L, Dall'Olio S, Beretti F, Portolano B, Russo V (2011). Coat colours in the Massese sheep breed are associated with mutations in the agouti signalling protein (ASIP) and melanocortin 1 receptor (MC1R) genes. Animal.

[bib1.bib17] Geist V (1966). The Evolutionary Significance of Mountain Sheep Horns. Evolution.

[bib1.bib18] Hepp D, Gonçalves GL, Moreira GR, Freitas TR, Martins CT, Weimer TA, Passos DT (2012). Identification of the e allele at the Extension locus (MC1R) in Brazilian Creole sheep and its role in wool color variation. Genet Mol Res GMR.

[bib1.bib19] Johnston SE, Mcewan JC, Pickering NK, Kijas JW, Beraldi D, Pilkington JG, Pemberton JM, Slate J (2011). Genome-wide association mapping identifies the genetic basis of discrete and quantitative variation in sexual weaponry in a wild sheep population. Mol Ecol.

[bib1.bib20] Kalds P, Zhou S, Gao Y, Cai B, Huang S, Chen Y, Wang X (2022). Genetics of the phenotypic evolution in sheep: a molecular look at diversity-driving genes. Genet Sel Evol.

[bib1.bib21] Klungland H, Vage DI, Gomez-Raya L, Adalsteinsson S, Lien S (1995). The role of melanocyte-stimulating hormone (MSH) receptor in bovine coat color determination. Mamm Genome.

[bib1.bib22] Koseniuk A, Ropka-Molik K, Rubiś D, Smołucha G (2018). Genetic background of coat colour in sheep. Arch Anim Breed.

[bib1.bib23] Li MH, Tiirikka T, Kantanen J (2014). A genome-wide scan study identifies a single nucleotide substitution in ASIP associated with white versus non-white coat-colour variation in sheep (Ovis aries). Heredity.

[bib1.bib24] Li Y, Zhang X, Pang Y, Qi Y, Zhao S (2019). Construction of MC1R and ASIP Eukaryotic Expression Vector and its Regulation of Plumage Color in Japanese Quail (Coturnix japonica). J Poult Sci.

[bib1.bib25] Liu N, He JN, Yu WM, Liu KD, Cheng M, Liu JF, He YH, Zhao JS, Qu XX (2015). Transcriptome analysis of skeletal muscle at prenatal stages in Polled Dorset versus Small-tailed Han sheep. Genet Mol Res GMR.

[bib1.bib26] Mandal BK, Chen H, Si Z, Hou X, Yang H, Xu X, Wang J, Wang C (2020). Shrunk and scattered black spots turn out due to MC1R knockout in a white-black Oujiang color common carp (Cyprinus carpio var. color). Aquaculture.

[bib1.bib27] Meng YC, Lin T, Jiang H, Zhang Z, Shu L, Yin J, Ma X, Wang C, Gao R, Zhou X (2020). miR-122 Exerts Inhibitory Effects on Osteoblast Proliferation/Differentiation in Osteoporosis by Activating the PCP4-Mediated JNK Pathway. Mol Ther-Nucl Acid.

[bib1.bib28] Parsons YM, Fleet MR, Cooper DW (1999). The Agouti gene: a positional candidate for recessive self-colour pigmentation in Australian Merino sheep. Aust J Agric Res.

[bib1.bib29] Price AL, Zaitlen NA, Reich D, Patterson N (2010). New approaches to population stratification in genome-wide association studies. Nat Rev Genet.

[bib1.bib30] Pan Z, Li S, Liu Q, Wang Z, Zhou Z, Di R (2018). Whole-genome sequences of 89 Chinese sheep suggest role of RXFP2 in the development of unique horn phenotype as response to semi-feralization. GigaScience.

[bib1.bib31] Rochus CM, Sunesson KW, Jonas E, Mikko S, Johansson AM (2019). Mutations in ASIP and MC1R: dominant black and recessive black alleles segregate in native Swedish sheep populations. Anim Genet.

[bib1.bib32] Shil S, Joshi RS, Joshi CG, Patel AK, Shah RK, Patel N, Jakhesara SJ, Kundu S, Reddy B, Koringa PG, Rank DN (2017). Transcriptomic comparison of primary bovine horn core carcinoma culture and parental tissue at early stage. Vet World.

[bib1.bib33] Smit MA, Shay TL, Beever JE, Notter DR, Cockett NE (2002). Identification of an agouti-like locus in sheep. Anim Genet.

[bib1.bib34] Simon R, Drögemüller C, Lühken G (2022). The Complex and Diverse Genetic Architecture of the Absence of Horns (Polledness) in Domestic Ruminants, including Goats and Sheep. Genes.

[bib1.bib35] Våge DI, Klungland H, Lu D, Cone RD (1999). Molecular and pharmacological characterization of dominant black coat color in sheep. Mamm Genome.

[bib1.bib36] Waldmann P, Mészáros G, Gredler B, Fuerst C, Sölkner J (2013). Evaluation of the lasso and the elastic net in genome-wide association studies. Front Genet.

[bib1.bib37] Wang Y, Zhang C, Wang N, Li Z, Heller R, Liu R, Zhao Y, Han J, Pan X, Zheng Z, Dai X, Chen C, Dou M, Peng S, Chen X, Liu J, Li M, Wang K, Liu C, Lin Z, Chen L, Hao F, Zhu W, Song C, Zhao C, Zheng C, Wang J, Hu S, Li C, Yang H, Jiang L, Li G, Liu M, Sonstegard TS, Zhang G, Jiang Y, Wang W, Qiu Q (2019). Genetic basis of ruminant headgear and rapid antler regeneration. Science.

[bib1.bib38] Wiedemar N, Drögemüller C (2015). A 1.8-kb insertion in the 3
${}^{\prime }$
-UTR of RXFP2 is associated with polledness in sheep. Anim Genet.

[bib1.bib39] Wiedemar N, Tetens J, Jagannathan V, Menoud A, Neuenschwander S, Bruggmann R, Thaller G, Drögemüller C (2014). Independent Polled Mutations Leading to Complex Gene Expression Differences in Cattle. PLOS ONE.

[bib1.bib40] Xiao J, Wu Y, Chen R, Lin Y, Wu L, Tian W, Liu L (2008). Expression of Pcp4 gene during osteogenic differentiation of bone marrow mesenchymal stem cells in vitro. Mol Cell Biochem.

[bib1.bib41] Yang GL, Fu DL, Lang X, Wang YT, Cheng SR, Fang SL, Luo YZ (2013). Mutations in MC1R Gene Determine Black Coat Color Phenotype in Chinese Sheep. Sci World J.

[bib1.bib42] Zhou X, Stephens M (2012). Genome-wide efficient mixed-model analysis for association studies. Nat Genet.

