# Supplementary material for: Genome-wide association study for polledness, horn shape, and wool traits in Original Valachian sheep
Source: Arch Anim Breed. 2024 Jul 30;67(3):373–81. doi: 10.5194/aab-67-373-2024 (PMC13189279; doi:10.5194/aab-67-373-2024)
Supplement: The supplement related to this article is available online at: https://doi.org/10.5194/aab-67-373-2024-supplement. [file aab-67-373-2024-supplement.pdf]

Supplement of Arch. Anim. Breed., 67, 373–381, 2024
https://doi.org/10.5194/aab-67-373-2024-supplement
© Author(s) 2024. CC BY 4.0 License.

Open Access
Archives Animal Breeding

Supplement of

Genome-wide association study for polledness, horn shape, and wool traits in Original Valachian sheep

Mária Mészárosová et al.

Correspondence to: Nina Moravčíková (nina.moravcikova@uniag.sk)

The copyright of individual parts of the supplement might differ from the article licence.

Table S1. QTL content in the genomic regions of 1 Mb centered on the most significant SNP from the GWAS study (source: https://animalgenome.org/QTLdb/).

Phenotype	Chr	Position (Mb)	SNP name	-log10(p) from GWAS	QTL	Gene content
Polledness	6	93.37	Chr6:93370144	4.46	Milk protein percentage<br>Milk yield<br>Reactivity to humans	*CXCL13, CNOT6L, MRPL1, FRAS1*
Polledness	10	29.45	RXFP2_insert_L1	4.15	Horn type<br>Horn circumference<br>Horn length<br>Tail fat deposition	*ZAR1L, FRY, RXFP2, B3GLCT*
Polledness	10	30.91	OAR10_31042465.1	11.11	Horn type<br>Horn circumference<br>Horn length<br>Tail fat deposition	*USPL1, HMGB1, KATNAL1, UBL3, SLC7A1, MTUS2*
side-way turned horns vs polled	2	143.33	OAR2_143337545.1	4.98	Intermuscular fat weight	*SCN7A, SCN1A, SCN9A, TTC21B*
side-way turned horns vs polled	4	83.45	OAR4_88648994.1	4.96	No QTL	*YAE1, POU6F2, VPS41*
side-way turned horns vs polled	6	88.76	OAR6_88762364.1	6.12	Fecal egg count	*ADAMTS3, ANKRD17, ALB, AFM*
side-way turned horns vs polled	10	30.91	OAR10_31042465.1	9.89	Horn type	*USPL1, HMGB1, KATNAL1, UBL3, SLC7A1, MTUS2*
side-way turned horns vs polled	23	14.93	s43526.1	5.11	Body and carcass weight<br>Average daily gain<br>Foot angle	No protein coding genes
Backwards-curled horns vs polled	2	247.47	DU499350_422.1	5.08	Milk fat percentage<br>Carcass and body weight	*UBR4, IFFO2, ALDH4A1, TAS1R2, PAX7*

					Bone density	
Backwards-curled horns vs polled	4	103.41	s11139.1	5.34	Blood characteristics Pneumonia susceptibility	*DGKI, CREB3L2, AKR1D1, TRIM24, SVOPL*
Backwards-curled horns vs polled	10	31.29	s03052.1	5.91	Horn type Fecal egg count Carcass traits	*UBL3, SLC7A1, MTUS2, SLC46A3*
Backwards-curled horns vs polled	26	31.67	OAR26_31673849_X.1	5.32	Stature Carcass weight Udder attachment	*KCNU1, ZNF703, ERLIN2, PLPBP*
Backwards-curled horns vs polled	26	40.22	OAR26_40222433.1	5.46	Stature Body weight Vocalization traits	*RARB*
side-way turned vs backwards-curled horns	1	260.79	s52939.1	6.16	No QTL	*LCA5L, B3GALT5, IGSF5, PCP4, DSCAM*
side-way turned vs the backwards-curled horns	3	42.35	s73851.1	13.86	No QTL	*MEIS1*
side-way turned vs the backwards-curled horns	3	82.69	s12856.1	7.13	Body weight Average daily gain Milk yield	No protein coding genes
side-way turned vs the backwards-curled horns	10	0.01	OAR10_12531.1	8.51	No QTL	*PCDH20*
side-way turned vs the backwards-curled horns	10	29.45	RXFP2_insert_L1 29456050	6.85	Horn type Horn length Horn circumference Tail fat deposition	*ZAR1L, FRY, RXFP2, B3GLCT*
side-way turned vs the backwards-curled horns	17	14.11	Chr17:14112366	5.96	Fecal egg count	*FREM3, SMARCA5*
side-way turned vs the backwards-curled horns	22	43.75	oar3_OAR22_43747064	6.74	Carcass bone percentage Muscle density	*CHST15, OAT, NKX1-2, FAM53B, EEF1AKMT2,*

						ABRAXAS2, ZRANB1, CTBP2
side-way turned vs the backwards-curled horns	26	40.87	oar3_OAR26_40876522	6.16	Vocalisation behavior<br>Vocalisation during isolation box test<br>Vocalisation during corridor test	*THRB, NR1D2*
side-way turned vs the backwards-curled horns	26	42.44	oar3_OAR26_42437568	8.75	No QTL	*UBE2E2, ZNF385D*
wool colour (white vs black)	14	14.23	oar3_OAR14_14231948	6.13	Coat colour<br>Fecal egg count<br>Total lambs born	*ACSF3, CDH15, SLC22A31, ANKRD11, SPG7, RPL13, CPNE7, DPEP1, CHMP1A, CDK10, SPATA2L, ZNF276, VPS9D1, FANCA, SPIRE2, TCF25, MC1R, DEF8, DBNDD1, GAS8, SHCBP1, VPS35, ORC6, MYLK3*
wool colour (white vs black)	20	10.77	oar3_OAR20_10774806	6.30	Milk Yield	*BNIP5, KCTD20, PXT1, STK38, SRSF3, CDKN1A, RAB44, CPNE5, PPIL1, PI16, FGD2, MTCH1, PIM1,*

						TBC1D22B, TMEM217
wool colour (white vs black)	24	15.37	s05526.1	5.75	No QTL	*ABCC1, ABCC6*
